# A Systematic Review and Meta-Analysis on the Efficacy and Safety of Finerenone Therapy in Patients with Cardiovascular and Chronic Kidney Diseases in Type 2 Diabetes Mellitus

**DOI:** 10.7759/cureus.41746

**Published:** 2023-07-11

**Authors:** FNU Jyotsna, Kamran Mahfooz, Tirath Patel, FNU Parshant, Fnu Simran, Fnu Harsha, Fnu Neha, Dev Jyotishna, Dipesh Mishra, Sirjana Subedi, Mahima Khatri, Satesh Kumar, Giustino Varrassi

**Affiliations:** 1 Medicine, DR. B.R. (Bharatha Rathna) Ambedkar Medical College & Hospital, Bengaluru, IND; 2 Internal Medicine, Lincoln Medical Center, New York, USA; 3 Medical Student, American University of Antigua, St. John's, ATG; 4 Medicine, Jinnah Sindh Medical University, Karachi, PAK; 5 Medicine, Ghulam Muhammad Mahar Medical College, Sukkur, PAK; 6 Cardiology, TU (Tribhuvan University) Teaching Hospital, International Organization for Migration, Mohali, NPL; 7 Medicine, Chirayu National Hospital and Medical Institute, Kathmandu, NPL; 8 Medicine, University of Medicine and Health Sciences, Basseterre, NPL; 9 Medicine and Surgery, Dow University of Health Sciences, Karachi, PAK; 10 Medicine and Surgery, Shaheed Mohtarma Benazir Bhutto Medical College, Karachi, PAK; 11 Pain Medicine, Paolo Procacci Foundation, Rome, ITA

**Keywords:** meta-analysis, non-steroidal mineralocorticoid receptor antagonist, finerenone, cardiovascular disease, ckd, chronic kidney disease, diabetes

## Abstract

The purpose of this study is to assess the safety and efficacy of finerenone therapy in type 2 diabetes mellitus (T2DM) patients with cardiovascular and chronic renal diseases. This meta-analysis assesses the efficacy and safety of finerenone in the treatment of diabetic kidney disease (DKD). A comprehensive search of PubMed, Embase, and Google Scholar databases was performed to identify relevant randomized controlled trials (RCTs). To quantify the effects of finerenone, the analysis included the estimation of aggregated mean differences (MDs) and relative risks (RRs), as well as 95% confidence intervals (CIs). This meta-analysis included seven double-blind trials with patients suffering from chronic kidney disease (CKD) and T2D. Participants received finerenone or a placebo was assigned at random. The primary efficacy outcomes were cardiovascular mortality, non-fatal myocardial infarction, non-fatal stroke, hospitalization for heart failure, kidney failure, a sustained 57% decrease in the estimated glomerular filtration rate from baseline over four weeks, or renal death. Among the 39,995 patients included in the analysis, finerenone treatment was associated with a lower risk of cardiovascular and renal-related mortality compared to placebo (RR = 0.86 (0.80, 0.93), p = 0.0002; I-squared statistic (I^2^ ) = 0%) and (RR = 0.56 (0.17, 1.82), p = 0.34; I^2 ^= 0%). In addition, finerenone treatment was associated with a marginally reduced risk of serious adverse events (RR = 0.95 (0.92, 0.97), p = 0.0001; I^2 ^= 0%), although no significant difference in the overall risk of adverse events was observed between the two groups (RR = 1.00 (0.99, 1.01), p = 0.56; I^2 ^= 0%). This study's findings suggest that finerenone administration can reduce the risk of end-stage kidney disease, renal failure, cardiovascular mortality, and hospitalization. Patients with both T2DM and CKD are therefore advised to consider finerenone therapy.

## Introduction and background

Heart failure (HF) is not a single pathological condition, but rather a clinical syndrome characterized by cardinal symptoms (e.g., shortness of breath, ankle edema, and fatigue) and signs (e.g., high jugular venous pressure, pulmonary crackles, and peripheral edema). It is caused by structural or functional abnormalities in the heart, resulting in increased intraventricular pressures and reduced cardiac output at rest or during physical activity [1]. The global prevalence of HF is rising, affecting a substantial number of people, with a diagnosis rate of 1% to 2% in the general population and 5% to 10% among those 65 and elderly [2].

The categorization of HF patients according to the left ventricular ejection fraction (LVEF) is crucial, as it influences survival rates and treatment responses. The majority of clinical studies select patients based on their ejection fraction, making a distinction between HF with reduced ejection fraction (HFrEF) (LVEF 40%) and HF with preserved ejection fraction (HFpEF) (LVEF > 40%). The prevalence of HFpEF is on the rise and accounts for at least 50% of all cases of HF [3].

HF, type 2 diabetes mellitus (T2DM), and chronic kidney disease (CKD) are significant health concerns in the 21st century [1]. Cardiovascular disease (CVD) is the primary cause of comorbidity in individuals with T2DM and is responsible for a significant number of deaths, with diabetes increasing the risk of CVD by two to fourfold. Moreover, diabetes in patients with existing CVD is strongly associated with poor clinical outcomes [4]. Despite current interventions, patients with CKD and T2DM continue to suffer from substantial cardiorenal morbidity and mortality. The severity and stage of CKD increases the risk of renal failure progression and cardiovascular complications. Individuals with better-preserved estimated glomerular filtration rate (eGFR) have a greater lifetime risk of CVDs, such as HF, myocardial infarction, stroke, and cardiovascular death, compared to those with advanced kidney disease who are more likely to progress to dialysis [5].

Diabetes-related diabetic kidney disease (DKD) is the most common cause of CKD, afflicting between 30% and 40% of diabetics. As the global prevalence of diabetes is expected to increase, DKD has emerged as one of the most devastating complications and the leading cause of renal failure. Pathologically, the progression of diabetes-related CKD is characterized by distinct processes, including an increase in mesangial substrate, nodular lesions, and tubulointerstitial fibrosis [6]. In the management of diabetes and CKD, interdisciplinary therapies that target critical pathophysiological pathways, such as angiotensin-converting enzyme (ACE) inhibitors, sodium-glucose cotransporter-2 (SGLT2) inhibitors, and mineralocorticoid receptor antagonists (MRAs), have been proposed and proven efficacious. These therapies not only benefit patients with diabetes and CKD, but they also have an impact on cardiac physiology [6]. Emerging evidence suggests that MR overactivation plays a significant role in CKD progression and associated morbidity and mortality. It accelerates the progression of CKD and CVD by causing inflammation and fibrosis in the heart, kidneys, and blood vessels. In preclinical models of CKD in T2D, the pharmacological inhibition of the MR has been demonstrated to reduce albuminuria, kidney fibrosis, glomerular lesions, and inflammation, with beneficial cardiovascular effects. However, the risk of hyperkalemia associated with the use of MRAs has limited their evaluation and use for severe renal and cardiovascular outcomes [7]. Nonetheless, concomitant use of other medications can mitigate side effects, such as hyperkalemia, making MR antagonism an intriguing therapeutic strategy to slow down the progression of CKD [8].

Finerenone, a nonsteroidal, selective MRA with anti-inflammatory and anti-fibrotic properties, has demonstrated promise in experimental trials for renal diseases and CVDs [9]. Studies have demonstrated a significant reduction in the incidence of cardiovascular mortality, nonfatal myocardial infarction, nonfatal stroke, or hospitalization for HF (the primary composite outcome) in individuals treated with finerenone versus placebo. In addition, finerenone has been linked to a lower incidence of severe adverse events, including pneumonia, compared to placebo [10].

Few randomized controlled trials (RCTs) have investigated the efficacy and safety profile of finerenone in relation to CKD and cardiovascular outcomes in patients with T2DM to date. Due to differences in adverse effect profiles, existing studies have yielded contradictory findings. To address this deficiency, we conducted a comprehensive systematic review and meta-analysis to provide a precise evaluation of finerenone's role in patients with CKD and T2D. This meta-analysis is the first of its kind to compare the efficacy of finerenone versus placebo in patients with CKD and T2D.

This article was previously posted to the Authorea preprint server on November 27, 2022.

## Review

Methodology

The research was carried out following the guidelines set forth in the Preferred Reporting Items for Systematic Reviews and Meta-Analyses (PRISMA) statement [11].

Search Strategy and Study Selection

We conducted a comprehensive search strategy to identify relevant studies for this research. PubMed (Medline) and Cochrane databases were thoroughly searched from the inception of the study until August 1, 2022. In addition, we explored literature and preprints through ClinicalTrials.gov, Google Scholar, and Medrxiv. To create the search strategy, we utilized keywords and Medical Subject Headings (MESH) terms encompassing topics, such as “finerenone,” “non-steroidal mineralocorticoid receptor antagonist,” “non-steroidal MRA,” “cardiac outcomes,” “cardio renal outcomes,” “type 2 diabetes,” and “diabetes Mellitus.” We did not impose any restrictions or filters on the search results. Non-English text was translated using Google's translate service. Furthermore, we manually searched reviews to identify additional relevant studies. Two independent reviewers (MK and SK) screened titles, abstracts, and full texts of the studies. To eliminate duplicates, we imported the identified studies into Endnote X9 (Clarivate Analytics, USA).

Eligibility Criteria

The selection of studies included in this analysis was based on several criteria. First, the study population consisted of adults aged 18 years and above who had T2D and CKD. The exposure of interest was the maximum tolerated labeled dose of an angiotensin-converting enzyme inhibitor (ACEi) or angiotensin receptor blocker (ARB). A comparison was made between the exposure group and the non-finerenone group, which received either the standard of care or a placebo. The primary focus of interest was to evaluate the efficacy and adverse effects of finerenone. To conduct the meta-analysis, completed randomized clinical trials that met the eligibility criteria were included. Observational studies, case reports, and reviews were excluded during the screening process. Furthermore, studies involving non-human participants, children under the age of 18, or pregnant women were not considered for analysis.

Data Extraction

The process of data extraction involved retrieving specific data from the relevant studies, such as the first author, publication year, study type, follow-up duration, total number of patients, and patients who received finerenone. In addition to baseline characteristics, such as age, gender, and standard therapy, comorbidities, such as CVD, hypertension, and DM, were extracted for both groups. All safety and efficacy outcomes adopted the definitions of the study's investigators.

Efficacy Outcomes

The efficacy outcomes centered on primary indicators, including a composite of kidney failure, defined as a sustained decrease of at least 40% in the eGFR from baseline over at least four weeks, or mortality from renal causes. Kidney failure was additionally defined as end-stage kidney disease or an eGFR of less than 15 mL per minute per 1.73 m2, with end-stage kidney disease defined as the initiation of long-term dialysis (for at least 90 days) or kidney transplantation. In addition, nonfatal myocardial infarction, nonfatal stroke, and hospitalization for HF were included in the composite of cardiovascular causes of mortality. Secondary outcomes included death from any cause, hospitalization for any reason, changes in the urinary albumin-to-creatinine ratio from baseline to month 4, and a composite outcome of kidney failure, sustained decrease of at least 57% in eGFR from baseline (equivalent to doubling the serum creatinine level) maintained for at least four weeks, or death from renal causes.

Safety Outcomes

The evaluation of adverse events, central laboratory testing, and serum potassium and creatinine levels was performed to determine the safety of the drug. Adverse events that occurred during the treatment period were defined as those that began or worsened during the administration of finerenone or placebo or within three days of any temporary or permanent interruption.

Study Quality Assessment

In order to evaluate the quality of clinical trials, two researchers independently evaluated randomized trials using the revised Cochrane risk-of-bias tool [12]. The evaluation included random assignment of participants, selective reporting of results, and the presence of lacking data.

Statistical Analysis

Review Manager 5.4 (Cochrane Collaboration, UK) was used to conduct the statistical analysis. For dichotomous outcomes, relative risks (RRs) and 95% confidence intervals (CIs) were calculated, whereas for continuous outcomes, mean values and standard deviations were provided. The meta-analysis utilized a random-effects model with generic-inverse variance and continuous outcome functions to present aggregated RRs and weighted mean differences (WMDs). Results with p-values less than 0.05 are considered statistically significant. Using funnel plots, the publication bias for each outcome was visually evaluated. Using the I^2^ statistic, the percentage of heterogeneity between trials was calculated. A value of 25% for I^2^ indicated low heterogeneity, whereas a range of 25% to 50% indicated moderate heterogeneity, and a range of 50% or more indicated high heterogeneity. A sensitivity analysis was conducted to evaluate the contribution of each study to the overall pooled estimate owing to the observed high degree of heterogeneity in the study outcomes.

Ethical Approval

Since the analysis utilized publicly available data, no ethical approval from the review board was necessary.

Results

Study Selection

**Figure 1:**
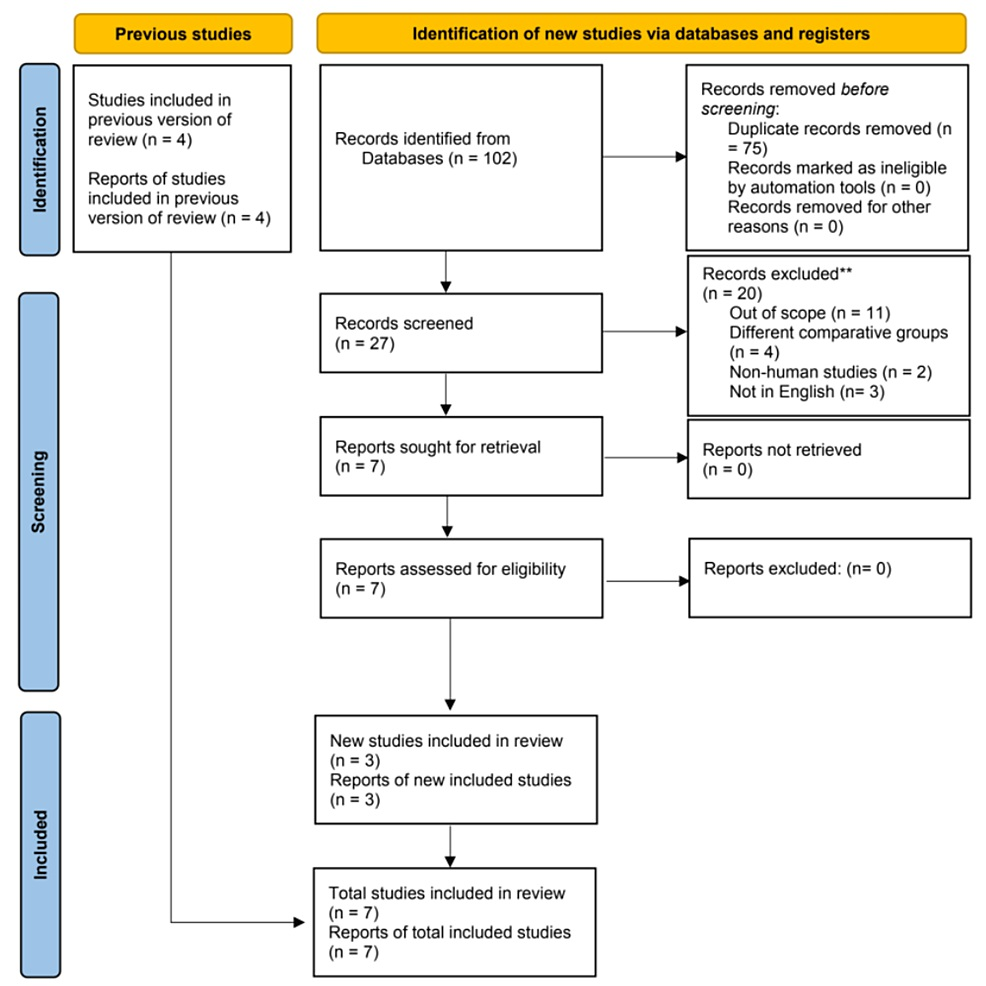
PRISMA flow diagram illustrating the search strategy and study selection process for the meta-analysis. PRISMA: Preferred Reporting Items for Systematic Reviews and Meta-Analyses

After conducting an initial search of the literature, a total of 46 articles were identified. Following the removal of duplicates and the evaluation of titles and abstracts, seven RCTs [5,10,13-17] were deemed suitable for inclusion in the meta-analysis. A summary of the study selection process can be found in Figure 1.

Baseline Characteristics

Among the 39,995 participants who met the inclusion criteria, a total of seven studies were included in the analysis. Of these participants, 20,368 (50.9%) received finerenone while 19,627 (49%) received a placebo. With the exception of Bakris (2015) [13] and Katayama (2017) [17], which were phase 2 trials, all other included trials were in phase 3. Additional details regarding the baseline characteristics of the participants can be found in Table 1, while Tables 2 and 3 provide information on comorbidities and previous medication history, respectively.

**Table 1:** Baseline characteristics of the included studies. SD: standard deviation; HBA1C: glycated hemoglobin; BMI: body mass index; No.: number of patients; N/A: not applicable

Study	Study design	Total no. of patients	No. of patients		Age (Mean±SD)		Male sex No. (%)		BMI kg/m^2^ (Mean±SD)		HBA1C % (Mean±SD)		Systolic blood pressure (Mean ±SD)		Diastolic blood pressure (Mean ±SD)		White No. (%)		Black No. (%)		Asians No. (%)		others No. (%)	
			Finerenone	Placebo	Finerenone	Placebo	Finerenone	Placebo	Finerenone	Placebo	Finerenone	Placebo	Finerenone	Placebo	Finerenone	Placebo	Finerenone	Placebo	Finerenone	Placebo	Finerenone	Placebo	Finerenone	Placebo
Bakris (2020) [13]	RCT	5674	2833	2841	65.4± 8.9	65.7±9.2	1953 (68.9)	2030 (71.5)	31.1 ± 6.0	31.1 ± 6.0	7.7± 1.3	7.7± 1.4	138.1±14.3	138.0± 14.4	75.8 ± 9.7	75.8 ± 9.7	1777 (62.7)	1815 (63.9)	140 (4.9)	124 (4.4)	717 (25.3)	723 (25.4)	199 (7.0)	179 (6.3)
Bertram (2021) [10]	RCT	7352	3686	3666	64.1± 9.7	64.1±10	2528 (68.6)	2577 (70.3)	31.5 ± 6.0	31.4 ± 5.9	7.7± 1.4	7.7± 1.4	135.8±14.0	135.7± 14.1	76.8 ± 9.5	76.8 ± 9.6	2672 (72.5)	2605 (71.1)	113 (3.1)	145 (4.0)	715 (19.4)	739 (20.2)	177 (4.8)	170 (4.6)
Gerasimos (2021) [14]	RCT	5674	2833	2841	65.5± 8.8	65.8±9	1953 (68.9)	2030 (71.4)	31.1±6	31.1±6	7.7± 1.3	7.7± 1.4	138± 14.3	138± 14.4	75.8± 9.7	75.8± 9.6	1717(60.6)	1815 (63.8)	140 (4.9)	124 (4.3)	717 (25.3)	723 (25.4)	N/A	N/A
Agarwal (2022) [5]	RCT	13026	6519	6507	64.7 ± 9.4	64.8 ± 9.7	4481 (68.7)	4607 (70.8)	N/A	N/A	7.7± 1.4	7.7 ± 1.4	136.8 ± 14.2	136.7± 14.3	76.3 ± 9.6	76.4 ± 9.6	4449 (68.2)	4420 (67.9)	253 (3.9)	269 (4.1)	1432 (22.0)	1462 (22.5)	385 (5.9)	356 (5.4)
Gerasimos (2022) [15]	RCT	7352	3686	3666	64.4± 9.34	65.1± 9.41	2528 (68.5)	2577 (70.2)	32.28± 6.2	31.84± 5.9	7.8± 1.4	7.79± 1.3	135.7± 13.7	135.3± 13.9	77.12± 9.6	76.41± 9.9	2672 (72.4)	2605 (71)	113 (3)	145 (3.9)	715 (19.3)	739 (20.1)	N/A	N/A

**Table 2:** Comorbidities of patients included in the study. N/A: not applicable; No.: number of patients

Study	Hypertension No. (%)		Diabetic retinopathy No. (%)		Diabetic neuropathy No. (%)		History of cardiovascular diseases No. (%)		Heart failure No. (%)		Peripheral arterial occlusive disease No. (%)		Ischemic stroke No. (%)	
	Finerenone	Placebo	Finerenone	Placebo	Finerenone	Placebo	Finerenone	Placebo	Finerenone	Placebo	Finerenone	Placebo	Finerenone	Placebo
Bakris (2015) [13]	685(94.2)	89 (94.7)	149(20.4)	19 (20.2)	139(19.1)	27(28.7)	N/A	N/A	N/A	N/A	N/A	N/A	N/A	N/A
Katayama (2017) [17]	84 (100)	12 (100)	40 (47.6)	5 (41.7)	30 (35.7)	6 (50)	15(17.8)	3(25)	N/A	N/A	N/A	N/A	N/A	N/A
Bakris (2020) [14]	2737 (96.6)	2768 (97.4)	1312 (46.3)	1351 (47.6)	738 (26.1)	716 (25.2)	1303 (46.0)	1302 (45.8)	195 (6.9)	241 (8.5)	470 (16.6)	453 (15.9)	329 (11.6)	360 (12.7)
Bertram (2021) [10]	3544 (96.1)	3517 (95.9)	1193 (32.4)	1098 (30.0)	1046 (28.4)	990 (27.0)	1676 (45.5)	1654 (45.1)	290 (7.9)	281 (7.7)	587 (15.9)	575 (15.7)	442 (12.0)	425 (11.6)
Gerasimos (2021) [15]	2737(96.6)	2768(97.3)	1312(46.3)	1351(47.5)	742(26.1)	722(25.4)	1303(45.9)	1302(45.8)	195 (6.8)	241(8.4)	N/A	N/A	N/A	N/A
Agarwal (2022) [5]	6281 (96.3)	6285 (96.6)	2505 (38.4)	2449 (37.6)	1788 (27.4)	1712(26.3)	2979 (45.7)	2956 (45.4)	485 (7.4)	522 (8.0)	1057 (16.2)	1028 (15.8)	771 (11.8)	785 (12.1)
Gerasimos (2022)[16]	3436(93.2)	3398(92.6)	N/A	N/A	N/A	N/A	1676(45.4)	1654(45.1)	290 (7.8)	281 (7.6)	N/A	N/A	N/A	N/A

**Table 3:** Medication history of the included patients. No.: number of patients; N/A: not applicable

Study	Study design	Total no. of patients	No. of patients		Age (Mean±SD)		Male sex No. (%)		BMI kg/m2 (Mean±SD)		HBA1C% (Mean±SD)		Systolic blood pressure (Mean ±SD)		Diastolic blood pressure (Mean ±SD)		White No. (%)		Black No. (%)		Asians No. (%)		others No. (%)	
			Finerenone	Placebo	Finerenone	Placebo	Finerenone	Placebo	Finerenone	Placebo	Finerenone	Placebo	Finerenone	Placebo	Finerenone	Placebo	Finerenone	Placebo	Finerenone	Placebo	Finerenone	Placebo	Finerenone	Placebo
Bakris (2020) [13]	RCT	5674	2833	2841	65.4± 8.9	65.7±9.2	1953 (68.9)	2030 (71.5)	31.1 ± 6.0	31.1 ± 6.0	7.7± 1.3	7.7± 1.4	138.1±14.3	138.0± 14.4	75.8 ± 9.7	75.8 ± 9.7	1777 (62.7)	1815 (63.9)	140 (4.9)	124 (4.4)	717 (25.3)	723 (25.4)	199 (7.0)	179 (6.3)
Bertram (2021) [10]	RCT	7352	3686	3666	64.1± 9.7	64.1±10	2528 (68.6)	2577 (70.3)	31.5 ± 6.0	31.4 ± 5.9	7.7± 1.4	7.7± 1.4	135.8±14.0	135.7± 14.1	76.8 ± 9.5	76.8 ± 9.6	2672 (72.5)	2605 (71.1)	113 (3.1)	145 (4.0)	715 (19.4)	739 (20.2)	177 (4.8)	170 (4.6)
Gerasimos (2021) [14]	RCT	5674	2833	2841	65.5± 8.8	65.8±9	1953 (68.9)	2030 (71.4)	31.1±6	31.1±6	7.7± 1.3	7.7± 1.4	138± 14.3	138± 14.4	75.8± 9.7	75.8± 9.6	1717(60.6)	1815 (63.8)	140 (4.9)	124 (4.3)	717 (25.3)	723 (25.4)	N/A	N/A
Agarwal (2022) [5]	RCT	13026	6519	6507	64.7 ± 9.4	64.8 ± 9.7	4481 (68.7)	4607 (70.8)	N/A	N/A	7.7± 1.4	7.7 ± 1.4	136.8 ± 14.2	136.7± 14.3	76.3 ± 9.6	76.4 ± 9.6	4449 (68.2)	4420 (67.9)	253 (3.9)	269 (4.1)	1432 (22.0)	1462 (22.5)	385 (5.9)	356 (5.4)
Gerasimos (2022) [15]	RCT	7352	3686	3666	64.4± 9.34	65.1± 9.41	2528 (68.5)	2577 (70.2)	32.28± 6.2	31.84± 5.9	7.8± 1.4	7.79± 1.3	135.7± 13.7	135.3± 13.9	77.12± 9.6	76.41± 9.9	2672 (72.4)	2605 (71)	113 (3)	145 (3.9)	715 (19.3)	739 (20.1)	N/A	N/A

Quality Assessment and Publication Bias

**Figure 2:**
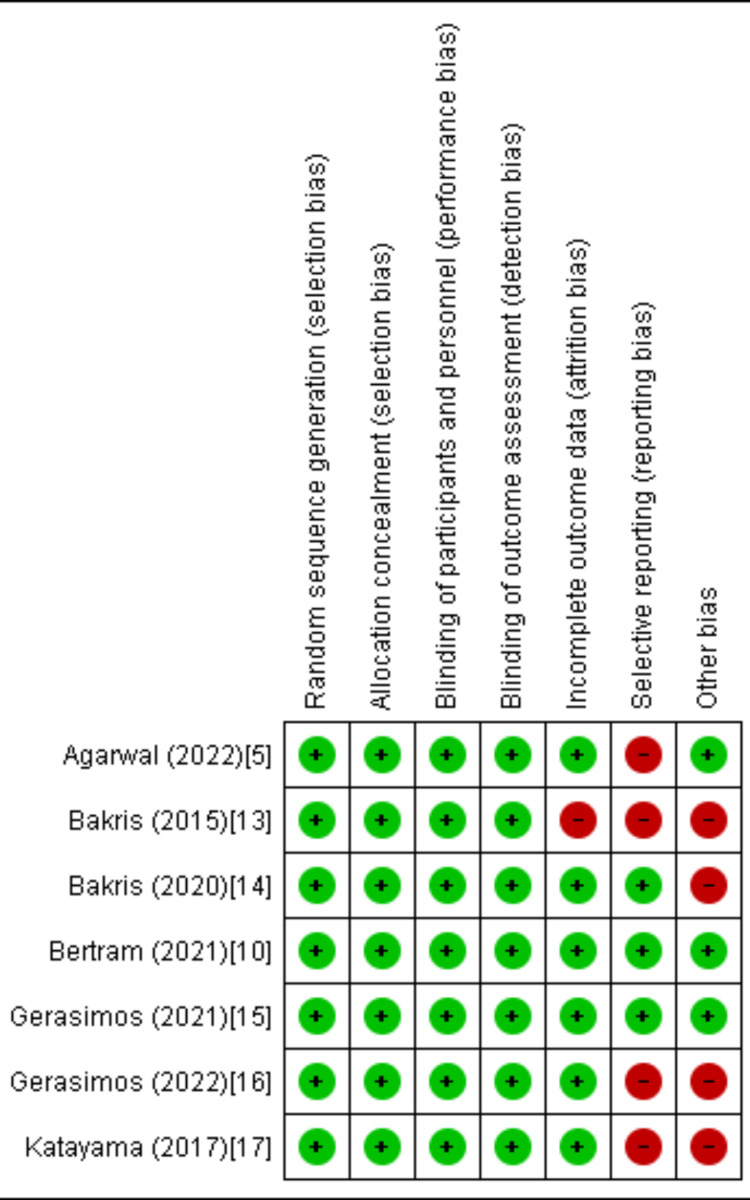
Quality assessment of the included randomized controlled trials. The trials of medium to high quality were identified using the Cochrane method for assessing randomized controlled trials (RCTs) [5,10,13-17].

**Figure 3:**
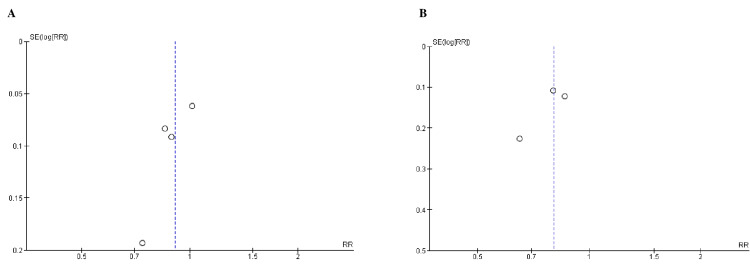
Funnel plots for A) kidney failure, B) ESRD (end-stage renal disease). RR (relative risk) was used as effect measures and SE (standard error) as a measure of precision. The funnel plots indicated no evidence of publication bias.

**Figure 4:**
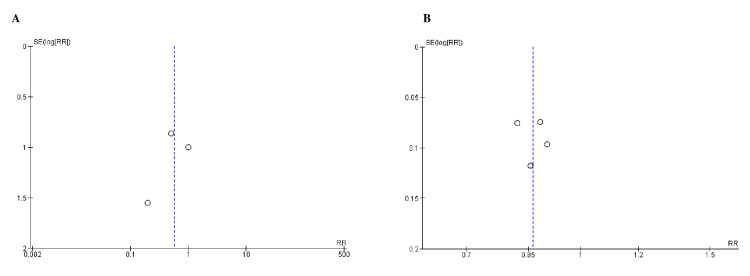
Funnel plots for A) sustained decrease to 15 mL/min/1.73 m2, B) sustained decrease of eGFR (estimated glomerular filtration rate) by >40% from baseline). RR (relative risk) was used as effect measures and SE (standard error) as a measure of precision. The funnel plots indicated no evidence of publication bias.

**Figure 5:**
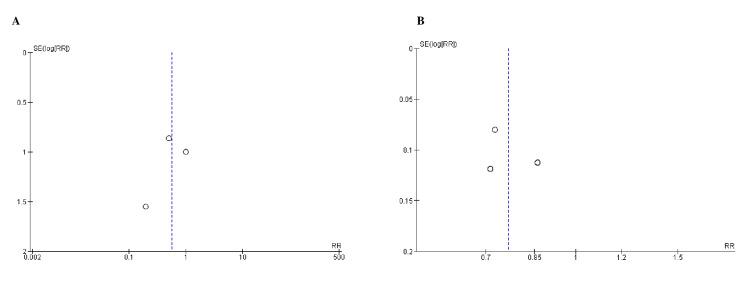
Funnel plots for A) death from renal causes and B) cardiovascular-related mortality. RR (relative risk) was used as effect measures and SE (standard error) as a measure of precision. The funnel plots indicated no evidence of publication bias.

**Figure 6:**
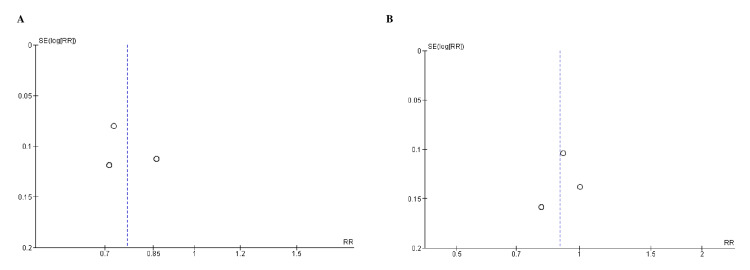
Funnel plots for A) hospitalization for heart failure and B) nonfatal myocardial infarction. RR (relative risk) was used as effect measures and SE (standard error) as a measure of precision. The funnel plots indicated no evidence of publication bias.

**Figure 7:**
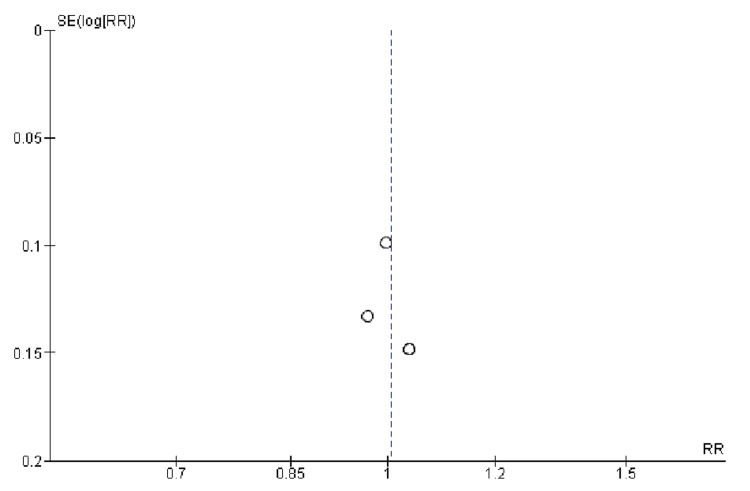
Funnel plot for nonfatal stroke. RR (relative risk) was used as effect measures and SE (standard error) as a measure of precision. The funnel plots indicated no evidence of publication bias.

Based on the Cochrane approach for assessing RCTs, we identified trials of satisfactory to excellent quality, as summarized in Figure 2. The funnel plots of the primary efficacy outcomes, as depicted in Figures 3, 4, 5, 6, and 7, demonstrated that the publication bias did not affect the findings.

Efficacy Outcomes

**Figure 8:**
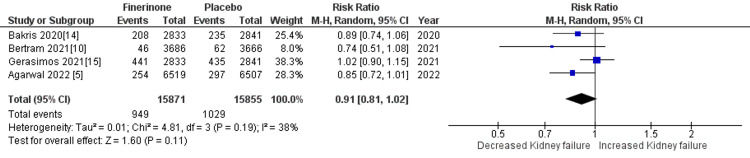
Forest plot for kidney failure. RR: relative risk; CI: confidence interval; M-H: Mantel Hansel [5,10,14,15]

**Figure 9:**
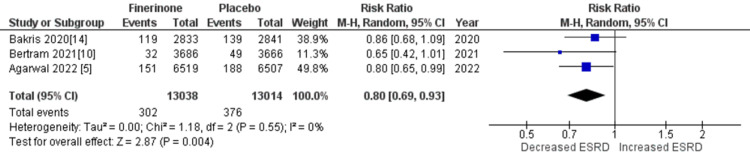
Forest plot for end-stage kidney disease. RR: relative risk; CI: confidence interval; M-H: Mantel Hansel [5,10,14,15]

**Figure 10:**
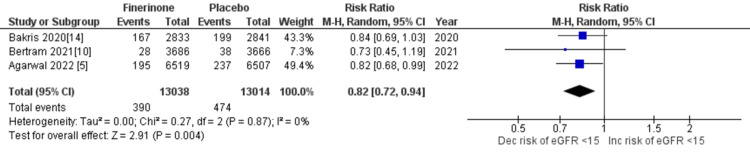
Forest plot of sustained decrease to 15 mL/min/1.73 m2. RR: relative risk; CI: confidence interval; M-H: Mantel Hansel [5,10,14,15]

**Figure 11:**
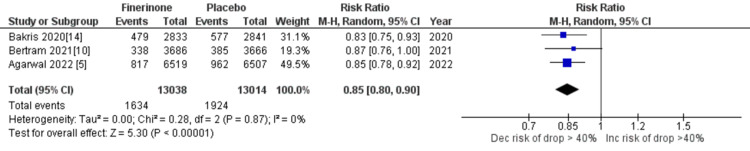
Forest plot showing sustained decrease of eGFR of >40% from baseline. eGFR: estimated glomerular filtration rate; RR: relative risk; CI: confidence interval; M-H: Mantel-Hansel [5,10,14]

**Figure 12:**
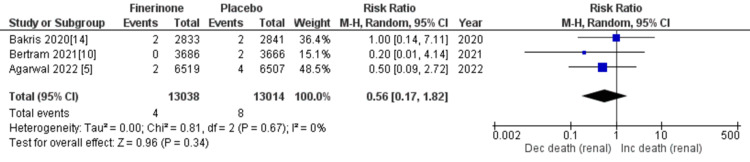
Forest plot showing death from renal causes. RR: relative risk; CI: confidence interval; M-H: Mantel-Hansel [5,10,14]

Composite of kidney failure: The composite measure for kidney failure encompassed several outcomes, including kidney failure, end-stage kidney disease, sustained decrease in eGFR to 15 mL/min/1.73 m2, sustained decrease of eGFR by more than 40% from baseline, and death from renal causes. Among the seven studies analyzed [5,10,14,15], four studies reported renal failure as an outcome. The pooled analysis demonstrated that treatment with finerenone was associated with a reduced risk of renal failure compared to placebo (RR = 0.91 (0.81, 1.01), p = 0.11; I^2^ = 38%), as shown in Figure 8. Similarly, three out of the seven studies [5,10,14] reported outcomes related to end-stage kidney disease, sustained decrease in eGFR to 15 mL/min/1.73 m2, sustained decrease of eGFR by more than 40% from baseline, and death from renal causes. The pooled analysis revealed that treatment with finerenone was associated with a decreased risk of these outcomes compared to placebo (RR = 0.80 (0.69, 0.93), p = 0.004; I^2^ = 0%), (RR = 0.82 (0.72, 0.94), p = 0.004; I^2^ = 0%), (RR = 0.85 (0.80, 0.90), p < 0.00001; I^2^ = 0%), and (RR = 0.56 (0.17, 1.82), p = 0.34; I^2^ = 0%), as depicted in Figures 9, 10, 11, and 12, respectively.

**Figure 13:**
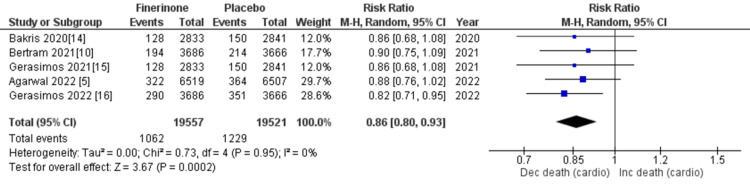
Forest plot showing death from cardiovascular causes. RR: relative risk; CI: confidence interval; M-H: Mantel-Hansel [5,10,14-16]

**Figure 14:**
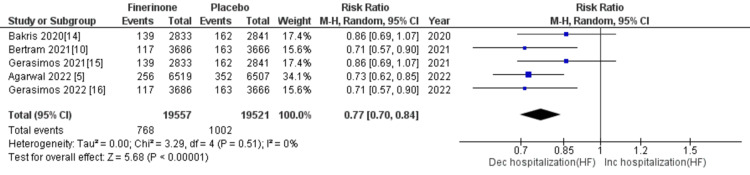
Forest plot showing the number of patients hospitalized for heart failure. RR: relative risk; CI: confidence interval; M-H: Mantel-Hansel [5,10,14-16]

**Figure 15:**
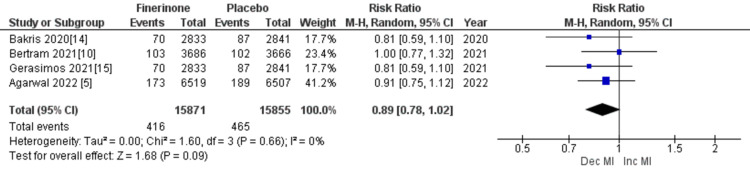
Forest plot showing the rate of nonfatal MI (myocardial infarction). RR: relative risk; CI: confidence interval; M-H: Mantel-Hansel [5,10,14,15]

**Figure 16:**
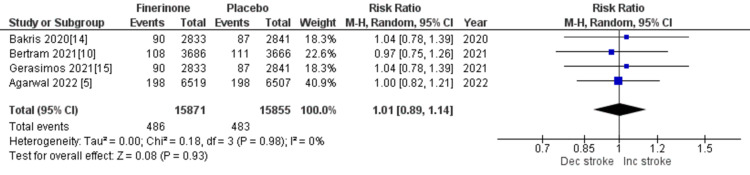
Forest plot showing the rate of nonfatal stroke. RR: relative risk; CI: confidence interval; M-H: Mantel-Hansel [5,10,14,15]

Composite cardiovascular outcomes: The composite outcomes related to cardiovascular events included cardiovascular-related mortality, hospitalization for HF, nonfatal myocardial infarction, and nonfatal stroke. Five out of seven studies [5,10,14-16] reported death from cardiovascular causes and hospitalization due to HF, and the combined analysis showed that treatment with finerenone was associated with a significant reduction in the risk of death and hospitalization as depicted in Figure 13 (RR = 0.86 (0.80, 0.93), p = 0.0002; I^2^ = 0%) and Figure 14 (RR = 0.77 (0.70, 0.84), p = 0.00001; I^2^ = 0%). Non-fatal myocardial infarction and non-fatal stroke were reported in four of the seven studies [5,10,14,15], and the pooled analysis indicated that treatment with finerenone was linked to a decreased risk of non-fatal myocardial infarction (RR = 0.89 (0.78, 1.02), p = 0.09; I^2^ = 0%), as shown in Figure 15. Meanwhile, there was no significant difference in the risk of non-fatal stroke between the two treatment groups (RR = 1.01 (0.89, 1.14), p = 0.93; I^2^ = 0%), as demonstrated in Figure 16.

 

Secondary outcomes: The secondary outcomes assessed in the study included mortality, hospitalization for any cause, and sustained decrease of 57% or more in eGFR from baseline. Out of the seven studies included [5,10,14], three studies reported all of these outcomes. The pooled analysis demonstrated that the treatment with finerenone was associated with a reduced risk of these outcomes: (RR = 0.90 (0.83, 0.97), p = 0.006; I^2^ = 0%), (RR = 0.97 (0.94, 0.99), p = 0.02; I^2^ = 0%), and (RR = 0.71 (0.64, 0.79), p < 0.00001; I^2^ = 0%).

Safety Outcomes

Adverse events: Three outcomes were employed to assess adverse events: the total number of patients experiencing any adverse event, adverse events related to the treatment regimen, and adverse events leading to discontinuation of the trial regimen. All seven studies [5,10,13-17] reported the total number of patients experiencing adverse events, and the pooled analysis demonstrated that the occurrence of any adverse event was comparable between the two groups (RR = 1.00 (0.99, 1.01), p = 0.56; I^2^ = 0%). However, adverse events related to the treatment regimen were reported in five out of seven studies [5,10,14,15,16], and the combined analysis revealed that treatment with finerenone was associated with an elevated risk of adverse events related to the treatment regimen (RR = 1.40 (1.33, 1.46), p < 0.00001; I^2^ = 0%). Likewise, adverse events leading to the discontinuation of the treatment regimen were reported in six out of seven studies [5,10,13-16], and the pooled analysis indicated that treatment with finerenone was linked to a higher risk of treatment discontinuation compared to placebo (RR = 1.62 (0.84, 3.10), p = 0.15; I^2^ = 98%). Considering the significant heterogeneity observed for adverse events leading to treatment discontinuation, a sensitivity analysis was conducted, which demonstrated that excluding the study by Gerasimos (2021) [15] reduced heterogeneity and rendered the results statistically significant (RR = 1.22 (1.11, 1.33), p = 0.0001; I^2^ = 0%).

Serious adverse events: The evaluation of serious adverse events was based on three specific outcomes: the number of patients experiencing a serious adverse event, the number of serious adverse events related to the trial regimen, and the number of serious adverse events leading to trial discontinuation. Out of the seven studies analyzed [5,10,13-17], six studies reported these outcomes. The pooled analysis indicated that the number of patients experiencing serious adverse events was slightly lower in the finerenone group compared to the placebo group (RR = 0.95 (0.92, 0.97), p < 0.0001; I^2^ = 0%). Similarly, the risk of treatment discontinuation due to serious adverse events was marginally lower in the finerenone group compared to the placebo group (RR = 0.94 (0.83, 1.07), p= 0.38; I^2^ = 0%). However, the risk of serious adverse events related to the trial regimen was higher in patients treated with finerenone (RR = 1.36 (1.13, 1.64), p = 0.001; I^2^ = 0%).

Hyperkalemia: The assessment of hyperkalemia included three specific outcomes: investigator-reported hyperkalemia, hyperkalemia related to the trial regimen, and permanent discontinuation of the trial regimen due to hyperkalemia. Out of the five studies analyzed [5,10,14,15], four studies reported investigator-reported hyperkalemia. The pooled analysis indicated that treatment with finerenone was significantly associated with an increased risk of hyperkalemia (RR = 2.20 (1.90, 2.55), p = 0.00001; I^2^ = 77%). To address the high heterogeneity, a leave-one-out analysis was performed, and excluding Betram (2021) [10] reduced heterogeneity (RR = 2.03 (1.89, 2.18), p < 0. 00001; I^2^ = 0%). Meanwhile, out of the seven studies analyzed [5,10,13-17], five studies reported hyperkalemia related to the trial regimen. The pooled analysis revealed that treatment with finerenone was significantly associated with an increased risk of hyperkalemia related to the treatment regimen. Similarly, out of the seven studies, six studies reported permanent discontinuation of the treatment regimen due to hyperkalemia. The pooled analysis showed that treatment with finerenone was significantly associated with a higher risk of treatment discontinuation due to hyperkalemia compared to placebo (RR = 2.59 (1.92, 3.50), p = 0.00001; I^2^ = 40%).

Serious hyperkalemia: Two outcomes, serious hyperkalemia and hyperkalemia-related hospitalization, were examined to evaluate the occurrence of severe hyperkalemia. These outcomes were reported in five out of seven studies [5,10,14-16]. The pooled analysis demonstrated that treatment with finerenone was significantly associated with an increased risk of serious hyperkalemia (RR = 4.25 (3.11, 5.83), p = 0.00001; I^2^ = 0%) and hospitalization due to hyperkalemia (RR = 5.94 (4.04, 8.75), p = 0.00001; I^2^ = 0%).

Hypokalemia: Investigator-reported hypokalemia was reported in three out of the seven studies [5,10,14], and the pooled analysis demonstrated that treatment with finerenone was significantly associated with a reduced risk of hypokalemia compared to placebo (RR = 0.47 (0.38, 0.57), p = 0.00001; I^2^ = 0%).

Renal-related adverse events: Renal-related adverse events were assessed using several outcomes, including acute kidney injury (AKI), hospitalization due to AKI, discontinuation of the trial regimen due to AKI, and hospitalization due to acute renal failure (ARF). Four out of the seven studies [5,10,14,15] reported on AKI, and the pooled analysis indicated that treatment with finerenone was associated with a slightly reduced risk of AKI (RR = 0.94 (0.84, 1.05), p = 0.30; I^2^ = 0%). Hospitalization due to AKI and hospitalization due to ARF were reported in three out of the seven studies [5,10,14], and the pooled analysis revealed that the risk of hospitalization for both outcomes was similar between the treatment groups: (RR = 0.99 (0.80, 1.22), p = 0.91; I^2^ = 0%) and (RR = 0.97 (0.80, 1.18), p = 0.76; I^2^ = 0%), respectively. However, the risk of discontinuation of the trial regimen due to AKI was higher in the finerenone group (RR = 1.38 (0.69, 2.75), p = 0.37; I^2^ = 0%).

Adverse events affecting ≥5% of patients in either group: Adverse events, such as hyperkalemia, nasopharyngitis, arthralgia, back pain, urinary tract infection, diarrhea, anemia, hypertension, upper respiratory tract infection, peripheral edema, decreased glomerular filtration rate (GFR), hypoglycemia, dizziness, bronchitis, constipation, and pneumonia, were observed in at least 5% of patients in both treatment groups. Patients receiving finerenone had a higher risk of experiencing hyperkalemia, anemia, decreased eGFR, dizziness, upper respiratory tract infection, diarrhea, and arthralgia. Conversely, they had a lower risk of developing hypoglycemia, pneumonia, peripheral edema, constipation, urinary tract infection, bronchitis, nasopharyngitis, and hypertension. Further information on adverse events affecting at least 5% of the study population can be found in Table 4.

**Table 4:** Adverse events affecting >/= 5% of patients in either group. CI: confidence interval; eGFR: estimated glomerular filtration rate

Adverse event	Risk ratio	95% CI	P value
Hyperkalemia	2.07	1.94-2.21	<0.00001
Nasopharyngitis	0.97	0.90-1.04	0.35
Hypertension	0.73	0.68-0.79	<0.00001
Anemia	1.07	0.99-1.17	0.10
Diarrhea	1.02	0.94-1.11	0.66
Upper respiratory tract infection	1.02	0.93-1.11	0.69
Decline in eGFR	1.30	1.15-1.47	<0.0001
Urinary tract infection	0.97	0.88-1.06	0.51
Back pain	1.01	0.93-1.10	0.74
Peripheral edema	0.65	0.60-0.70	<0.00001
Hypoglycemia	0.87	0.77-0.99	0.04
Dizziness	1.04	0.94-1.414	0.43
Arthralgia	1.06	0.97-1.15	0.17
Bronchitis	0.97	0.88-1.06	0.49
Constipation	0.91	0.80-1.03	0.14
Pneumonia	0.70	0.64-0.77	<0.00001

Discussion

Diabetes is the primary cause of CKD and increases the risk of progressing to end-stage renal disease. Patients with diabetes and CKD face a significantly higher risk of CVD, which is the leading cause of illness and death [18]. Several well-established risk factors contribute to the development of DKD, including activation of the renin-angiotensin-aldosterone system (RAAS), hypertension, hyperglycemia, dyslipidemia, and proteinuria. To address these risks, ACEis, ARBs, and renin inhibitors are commonly prescribed to patients. Although these medications slow down the decline in renal function, they do not halt it completely [19]. Recent evidence suggests that the MR signaling pathway plays a role in renal damage independent of angiotensin II. As a result, MRAs, such as finerenone, have emerged as a promising approach to slow the progression of CKD in patients with residual kidney disease [20,21].

Studies have shown that finerenone, a new MRA, has clinically significant effects on improving renal outcomes and reducing cardiovascular mortality and morbidity in patients with CKD and T2D [18]. For instance, the Finerenone in Reducing Kidney Failure and Disease Progression in Diabetic Kidney Disease (FIDELIO-DKD) study demonstrated that finerenone decreases the likelihood of new-onset atrial fibrillation or flutter, as well as the risk of kidney or cardiovascular events, regardless of underlying arrhythmia history. Similarly, the FIGARO-DKD trial revealed that finerenone substantially reduces the occurrence of HF outcomes and lowers the risk of hospitalization for HF compared to placebo in T2D and CKD patients without symptomatic HFrEF [15,16].

In the overall population, patients receiving finerenone therapy had a lower risk of cumulative kidney outcomes, such as renal failure, sustained drop in eGFR, and mortality from renal causes, compared to those receiving placebo. Phase 2 trials (Mineralocorticoid Receptor Antagonist Tolerability Study (ARTS), ARTS-Diabetic Nephropathy (ARTS-DN), and ARTS-Heart Failure (ARTS-HF)) also demonstrated that finerenone was associated with a significantly lower risk of kidney failure, sustained eGFR drop, or renal death compared to placebo in diabetic patients with albuminuria taking ACE inhibitors or ARBs [22]. However, the phase 3 FIGARO-DKD trial did not show a significant difference in the incidence of composite endpoints between finerenone and placebo groups [10].

Regarding cardiovascular outcomes, major studies have reported a decrease in cardiovascular deaths, hospitalizations due to HF, and non-fatal myocardial infarction with finerenone therapy. However, no significant reduction in non-fatal stroke was observed compared to placebo. In a prototype of post-myocardial infarction HF, finerenone demonstrated therapeutic efficacy in enhancing both systolic and diastolic left ventricular functions, cardiac contractility, and relaxation. It also reduced pro-B-type natriuretic peptide levels without affecting blood pressure [23]. The ARTS-HF trial revealed that finerenone had comparable efficacy to eplerenone in reducing N-terminal pro-B-type natriuretic peptide (NT-proBNP) levels and showed a lower likelihood of adverse events, such as all-cause mortality, cardiovascular hospitalization, or emergency presentation for worsening HF [24].

Across the studies, the incidence of adverse events during treatment was similar between finerenone and placebo, but discontinuation due to the trial regimen was more common in the treatment group [5,10,13-17]. In addition, compared to spironolactone, finerenone had a lower incidence of hyperkalemia but similar effects on NT-proBNP and albuminuria [25]. While eplerenone is considered a viable alternative to spironolactone, finerenone has emerged as a potential substitute with a reduced risk of hyperkalemia and comparable cardiovascular and renal benefits to other MRAs [25]. Finerenone is generally well tolerated, has a safe pharmacological profile, and exhibits predictable dose-dependent effects [25]. Furthermore, finerenone therapy was associated with a lower risk of hypokalemia compared to placebo.

In our study, we observed that the worsening of HF in patients leads to varying degrees of compromised kidney function. The use of potassium-sparing diuretics increases the risk of hyperkalemia in congestive HF patients receiving MRA therapy with renal dysfunction. Therefore, monitoring serum potassium levels is crucial. Compared to spironolactone or eplerenone, finerenone has fewer adverse effects on blood potassium levels and eGFR [26]. Although finerenone therapy was associated with a slight reduction in the risk of AKI, there was no change in the risk of hospitalization due to AKI and ARF [27]. Recent data support the potential of MR antagonism in preventing the acute and long-term consequences of renal ischemia/reperfusion and slowing CKD progression [27].

Our meta-analysis offers several advantages. First, the inclusion of a larger sample size compared to previous meta-analyses enhances the credibility of our findings. Second, we employed various statistical tests and plots to assess publication biases, and the results indicated no publication bias. Lastly, sensitivity analysis was conducted to evaluate the impact of heterogeneous studies on the pooled estimate. However, our analysis does have some limitations. The follow-up periods varied among the included studies, with some having longer durations, and long-term follow-ups are essential for evaluating the efficacy of finerenone therapy in chronic conditions like CKD and T2D. Furthermore, different doses of finerenone were used in various studies, and some studies lacked control groups or reported doses, which introduced uncertainty. In addition, half of the studies involving CKD and T2D patients did not report vital renal and cardiovascular outcomes, such as the number of patients with end-stage kidney disease and deaths from renal and cardiovascular causes.

## Conclusions

Based on our comprehensive analysis of various studies, the use of finerenone therapy has demonstrated positive effects on both renal and cardiovascular outcomes. This includes a notable decrease in the risk of end-stage kidney disease, renal failure, and a significant reduction in death and hospitalization due to cardiovascular issues. The therapy's favorable therapeutic profile also allows it to effectively address fluctuations in potassium levels. As a result, we highly recommend the utilization of finerenone therapy in patients who have both T2DM and CKD. However, it is important to note that further RCTs of adequate quality are necessary to explore the additional benefits of finerenone therapy, specifically in CKD patients with T2DM.
